# Myocardial inflammation in myocarditis: mechanisms and therapeutic targets

**DOI:** 10.3389/fcvm.2026.1821475

**Published:** 2026-06-23

**Authors:** Cristina Madaudo, Daniela Di Lisi, Francesca Macaione, Vincenzo Nuzzi, Andrea Micarelli, Giusy Sausa, Giovanni Castelli, Egle Corrado, Alfredo Ruggero Galassi, Giuseppina Novo

**Affiliations:** 1 Department of Health Promotion, Mother and Child Care, Internal Medicine and Medical Specialties (ProMISE), University of Palermo, Palermo, Italy; 2 Division of Cardiology, University Hospital P. Giaccone, University of Palermo, Palermo, Italy; 3 Department of Clinical Cardiology and Heart Failure, Mediterranean Institute for Transplantation and Advanced Specialized Therapies, IRCCS ISMETT, UPMC, Palermo, Italy

**Keywords:** immunomodulatory therapy, inflammation, inflammation-guided risk stratification, innate and adaptive immunity, myocarditis

## Abstract

Myocarditis is an inflammatory disease of the myocardium with heterogeneous aetiologies, in which innate and adaptive immune responses critically influence clinical outcomes, ranging from complete recovery to progression toward dilated cardiomyopathy. In viral myocarditis, pathogen- and damage-associated molecular patterns activate pattern-recognition receptors, triggering NF-*κ*B and inflammasome signaling and amplifying pro-inflammatory cytokine responses. Early myocardial injury involves neutrophils, mast cells, natural killer cells, and inflammatory monocytes, followed by activation of adaptive immune responses. Imbalance of Th1/Th2 immunity and dysregulation of the Th17/Treg axis promote persistent inflammation and fibrotic remodeling. Autoimmune mechanisms driven by molecular mimicry, pathogenic autoantibodies, and genetic susceptibility further sustain myocardial injury. While conventional biomarkers support diagnosis, emerging inflammatory markers and immune-derived indices show promise for risk stratification and disease monitoring. Beyond guideline-directed heart failure therapy, targeted immunomodulatory strategies are under investigation, highlighting the need for precision approaches to prevent chronic inflammation and adverse remodeling.

## 1 Introduction

**Figure 1:**
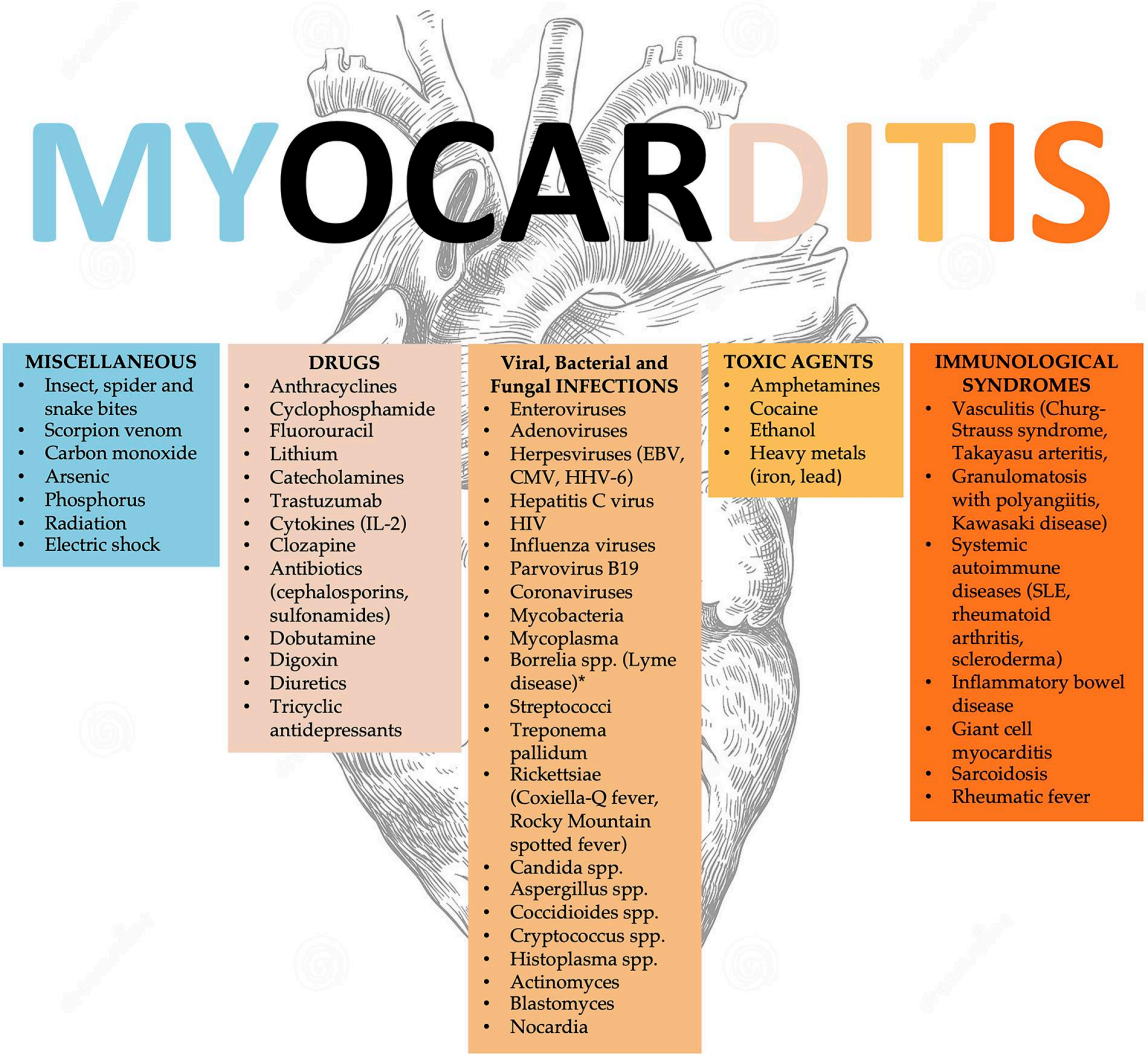
Schematic overview of the main infectious, immune-mediated, toxic, and drug-related causes of myocarditis, illustrating the heterogeneity of etiological mechanisms contributing to myocardial inflammation. CMV, cytomegalovirus; EBV, epstein–barr virus; HHV-6, human herpesvirus 6; HIV, human immunodeficiency virus; IL-2, interleukin-2; SLE, systemic lupus erythematosus; SPP., species pluralis (indicates multiple species within the same genus).

Myocarditis is a heterogeneous inflammatory disease of the myocardium characterized by highly variable clinical presentations and outcomes, ranging from spontaneous recovery to progressive inflammatory cardiomyopathy and heart failure (HF) (1, 2). (Figure 1). Although major advances have improved the understanding of myocarditis pathogenesis, the mechanisms linking immune activation, myocardial injury, and adverse remodeling remain incompletely understood. Increasing evidence suggests that dysregulated inflammatory responses, rather than direct pathogen-mediated injury alone, play a central role in determining disease severity and progression. In this context, inflammation has emerged not only as a key pathogenic driver, but also as a potential tool for risk stratification and therapeutic targeting, supporting growing interest in biomarker-guided and mechanism-based approaches in myocarditis (3).

In recent years, research has increasingly focused on the immunopathological mechanisms of myocarditis, showing that an excessive or dysregulated inflammatory response is often the main driver of myocardial damage (4). This is particularly relevant in fulminant myocarditis, immune checkpoint inhibitor (ICI)-associated myocarditis, and inflammatory cardiomyopathies where immune-driven damage can be severe and potentially fatal (5). Understanding the immune mechanisms that mediate myocardial inflammation is therefore essential not only to refine diagnosis but also to guide treatment and identify patients at risk of adverse outcomes.

This review provides a focused overview of the inflammatory mechanisms underlying myocarditis, highlighting the roles of innate and adaptive immunity, key cytokine and inflammasome pathways, and the emerging relevance of novel biomarkers and therapeutic targets. Rather than offering an exhaustive discussion of diagnostic and imaging aspects, we specifically emphasize the pathobiology of inflammation and its clinical implications. In contrast to previous reviews, this work aims to bridge immunopathological insights with clinical translation, underscoring the potential of inflammation-guided risk stratification, biomarker-informed assessment, and emerging precision immunomodulatory strategies in myocarditis.

## 2 Etiological triggers of inflammation

### 2.1 Infective causes

Inflammatory myocardial injury can be broadly classified into infectious and non-infectious causes, with infectious etiologies, particularly viral myocarditis, being the most common (6). Viral genomes are frequently detected in endomyocardial biopsy (EMB) specimens from patients with myocarditis (7). Moreover, several studies have reported viral genomes in up to ∼70% of patients with idiopathic dilated cardiomyopathy (DCM) (8). A wide range of viruses can trigger myocardial inflammation. The most frequently implicated is parvovirus B19 (B19 V), followed by enteroviruses (e.g., coxsackieviruses and echoviruses) and adenoviruses. Other viruses include HHV-6, EBV, CMV, HCV, HIV, and influenza viruses (9). Pathogenetic mechanisms differ by viral tropism. Enteroviruses and adenoviruses are cardiotropic and can directly infect cardiomyocytes by binding the coxsackievirus and adenovirus receptor (CAR), inducing necrosis and/or apoptosis through viral replication. In persistent coxsackievirus infection, protease 2A, by targeting the cytoskeletal protein dystrophin, may contribute to the progression toward DCM (9, 10). In contrast, B19 V is primarily vasculotropic, infecting endothelial cells and promoting microvascular dysfunction, with secondary cardiomyocyte injury mediated by ischemia and sustained release of pro-inflammatory cytokines (9, 10). CMV, EBV, and HHV-6 are lymphotropic; myocardial damage is driven less by direct cytolysis and more by the host immune response to infection (9). Finally, HCV, HIV, and influenza A/B can promote myocardial injury predominantly through immune-mediated mechanisms, including cytokine storm–like inflammatory responses (10, 11).

### 2.2 Non-infective causes

Non-infectious myocarditis includes autoimmune forms, defined by immunohistochemical criteria for myocarditis in the absence of detectable viral genomes on PCR, with anti-heart antibodies (AHAs) either present or absent (12). Autoimmune myocarditis may occur as an isolated entity (e.g., giant cell myocarditis), as a post-infectious form, or in association with systemic autoimmune diseases such as rheumatoid arthritis (9), Systemic Lupus Erythematosus (SLE), Sjögren's syndrome, vasculitis, and polymyositis (13). Post-infectious autoimmunity may follow viral myocarditis via molecular mimicry, in which similarities between viral and cardiac antigens (including myosin) promote sustained inflammation and ongoing myocardial injury (6). Another mechanism is exposure of cryptic cardiac antigens secondary to virus-induced tissue damage; a prototypical example is cardiac myosin, particularly *α*-myosin heavy chain (*α*MyHC) (13).

## 3 Immunopathogenic mechanism

### 3.1 The triphasic model of viral myocarditis

**Figure 2:**
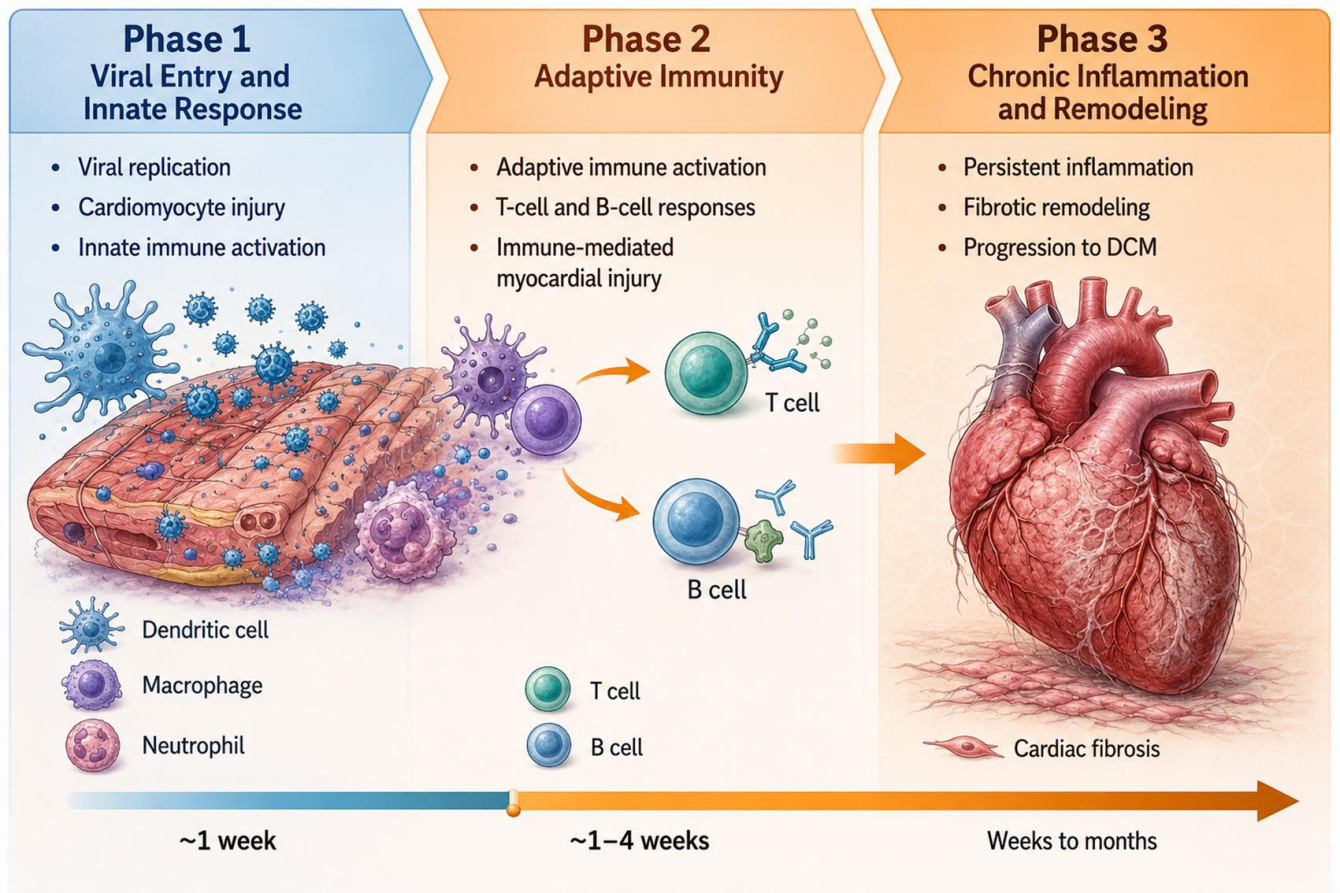
Schematic representation of the three-phase immunopathogenic model of viral myocarditis. The early phase is characterized by viral replication, cardiomyocyte injury, and activation of innate immune responses. This is followed by adaptive immune activation involving T- and B-cell responses, which may contribute to both viral clearance and immune-mediated myocardial injury. In susceptible individuals, persistent inflammation and immune dysregulation may drive fibrotic remodeling and progression toward dilated cardiomyopathy (DCM).

Viral myocarditis is commonly conceptualized as a dynamic three-phase process linking viral injury, immune activation, and chronic remodeling. The initial phase is characterized by viral replication, cardiomyocyte injury, and activation of innate immune responses aimed at pathogen clearance. This is followed by adaptive immune activation, which may contribute both to viral control and immune-mediated myocardial damage. In a subset of patients, unresolved inflammation and persistent immune dysregulation promote fibrotic remodeling and progression toward dilated cardiomyopathy (DCM) (6). Although the temporal boundaries are variable and often overlapping, the early phase generally occurs within the first week after infection, followed by adaptive immune activation over the subsequent weeks, whereas chronic inflammatory remodeling may persist for months (10) (Figure 2).

### 3.2 Role of PAMPs, DAMPs, PRRs

During the early phase, pathogen-associated molecular patterns (PAMPs) and damage-associated molecular patterns (DAMPs) are central to innate immune activation. DAMPs (also termed alarmins) comprise endogenous molecules released by dying or injured cells, including cardiomyocytes and immune cells (6). Examples include HMGB1, histones, S100 proteins, and heat shock proteins (HSPs) (14, 15). PAMPs and DAMPs activate inflammatory signaling through pattern recognition receptors (PRRs) expressed by innate immune cells (14). PRRs include membrane and cytosolic receptors. In viral myocarditis, Toll-like receptors (TLRs) induce production of inflammatory cytokines and type I interferons (16). Cytosolic receptors also contribute, including NLRP3 (a member of the NOD-like receptor family) required for the assembly of the inflammasome complex (17), and RIG-I–like receptors (RLRs) such as RIG-I and MDA5, which sense viral RNA and induce type I interferon responses (14). In murine models of CVB3 myocarditis, MDA5 deficiency has been associated with increased mortality, consistent with impaired type I interferon production (17).

A key downstream node is NF-*κ*B, a central transcription factor regulating inflammatory responses in both innate and adaptive immunity. NF-*κ*B promotes transcription of cytokines, chemokines, and adhesion molecules (16). PRR-driven NF-*κ*B activation induces TNF-α, IL-2, IFN-*γ*, pro–IL-1β, and pro–IL-18. IL-1β and IL-18 require inflammasome-mediated activation (17). Inflammasomes are cytosolic multiprotein complexes composed of a sensor, the adaptor ASC, and caspase-1 (17).

The NLRP3 inflammasome is notably upregulated in CVB3 myocarditis, and NLRP3 is a target of NF-*κ*B (17). Following priming, NLRP3 activation is promoted by disturbances in cellular homeostasis, including ionic fluxes, mitochondrial dysfunction, reactive oxygen species (ROS) generation, and lysosomal damage. Lysosomal disruption can activate NLRP3 through cytosolic acidification and release of proteases (e.g., calpain-1), which can further injure mitochondria and amplify oxidative stress. Cathepsin B has also been implicated in CVB3 myocarditis by enhancing caspase-1 activity and promoting myocardial pyroptosis (17).

### 3.3 Innate immune response

Multiple innate immune cell types infiltrate the myocardium, including natural killer (NK) cells (6), mast cells, neutrophils, dendritic cells, and monocytes/macrophages (10). Monocytes/macrophages are typically the dominant infiltrating population in both human myocarditis and experimental models (6, 15). Although the innate response is essential for pathogen clearance, excessive or persistent activation can promote tissue injury and contribute to disease progression (10, 17). Mast cells are among the earliest responders, releasing TNF-α, IL-1β, and IL-4 (10).

Neutrophils also contribute early in CVB3 murine models, becoming detectable within ∼2.5 days and accumulating rapidly (10). They recognize CVB3 largely through TLR8, and increased neutrophil activity correlates with greater myocardial injury, serving as a marker of disease severity (6). Neutrophil extracellular traps (NETs) can exacerbate injury via NETosis, a cytolytic process that releases large amounts of DAMPs and amplifies inflammation (6, 18). Early neutrophil infiltration, together with monocytes/macrophages, represents a major source of myocardial inflammatory cytokines (6). Neutrophils also release heparin-binding protein (HBP), which promotes endothelial cytoskeletal rearrangement, increases vascular permeability, and facilitates leukocyte extravasation (19).

Monocytes/macrophages play a central role in myocardial injury (10). Circulating monocytes include pro-inflammatory subsets with high CCR2 expression and less inflammatory subsets with lower CCR2 expression (10). Chemokines that recruit pro-inflammatory monocytes to the myocardium are produced by cardiac fibroblasts and are induced by IFN-*γ* released from infected cardiomyocytes. Key chemokines include CCL2 (MCP-1), CCL7, and MIP-1*α* (6, 10, 20). MCP-1/CCL2 and MIP-1*α* bind to CCR2 and CCR5, respectively, which are expressed on monocytes and activated T lymphocytes (20, 21). Infiltrating inflammatory monocytes can differentiate into M1 macrophages (10). These cells clear pathogens and debris and shape immune responses; however, if their activity is excessive or prolonged, it amplifies injury through the production of IL-1, TNF-α, IL-12, IL-23, and ROS (22). IFN-*γ*–driven activation (often in a Th1 context) enhances antigen presentation capacity (13). Cardiac fibroblasts contribute not only to monocyte recruitment but also to a microenvironment that favors maturation toward an M1 phenotype (10). In experimental autoimmune myocarditis, IL-17A levels rise in the acute phase and promote both monocyte migration and differentiation into classical macrophages. Mechanistically, IL-17A induces fibroblast production of MCP-1 and GM-CSF; GM-CSF, in turn, drives a pathological transcriptional program in monocytes, sustaining an inflammatory cascade and further myocardial injury (23). By contrast, M2 macrophages contribute to tissue repair and fibrosis. CCL24 has been proposed as a chemokine that may support monocyte recruitment and/or facilitate polarization toward M2 macrophages (6).

NK cells also participate in myocarditis. Cytotoxic NK subsets release granzymes and perforin to eliminate infected cells, whereas other subsets produce cytokines and chemokines such as TNF-α and MIP-1*α* (6). During viral myocarditis, IFN-*γ* induces CXCL10 expression of cardiomyocytes, thereby recruiting NK cells. In murine models, NKG2D signaling appears critical for CVB3 clearance and may limit progression to inflammatory cardiomyopathy (6).

Dendritic cells are potent antigen-presenting cells. They infiltrate the heart after viral infection in parallel with macrophage accumulation, phagocytose dying cardiomyocytes, and migrate to lymph nodes and spleen, where they initiate adaptive immune responses by activating antigen-specific CD4 + and CD8+ T cells (6).

### 3.4 Adaptive immune response

Multiple CD4+ T-cell lineages contribute to myocarditis, including Th1/Th2 and Th17/Treg axes. Th1 differentiation is driven by IL-12 and IFN-*γ*, and Th1 cells produce IFN-*γ* (6). IL-12 is produced by M1 macrophages and antigen-presenting cells (24). Th2 cells produce IL-4 and IL-10; although Th2 responses can attenuate viral acute myocarditis (AM), they may promote progression to inflammatory cardiomyopathy by stimulating fibrotic remodeling. Thus, an appropriate Th1/Th2 balance is important for limiting disease severity (6).

IL-12 has been implicated as a pro-inflammatory mediator in myocarditis (6). IL-12 receptors are expressed on activated NK cells and T lymphocytes; IL-12 signaling can increase IL-1β, IL-18, and TNF-α production, exacerbating myocardial injury (24). Conversely, IFN-*γ* appears protective (6, 24). IFN-*γ* promotes macrophage activation and viral clearance and may counteract fibrosis by inhibiting IL-4 production by Th2 cells and mast cells (6). IFN-*γ* also suppresses mast-cell activation, reduces fibroblast proliferation, and decreases collagen production (24). A key pro-inflammator*y* axis involves Th17 and regulatory T cells (Tregs) (6). A Th17-skewed phenotype is characterized by increased circulating Th17 cells, reduced Tregs, and elevated pro-inflammatory cytokines including IL-1β, IL-6, TGF-*β*, GM-CSF, and IL-23 (25). Cardiac myosin contributes to this phenotype: after release during myocardial injury, it can bind TLR2 on monocytes and stimulate production of these mediators (25). IL-6 and TGF-*β* (together with IL-21) activate Th17 differentiation via STAT3 and induce IL-23R expression. IL-23 produced by antigen-presenting cells promotes IL-17 production and maturation (6). IL-17 is a strong activator of NF-*κ*B and promotes expression of IL-1β, IL-18, IL-6, TNF-α, IL-12, IL-8, CCL2 (16, 26), and GM-CSF (23). These mediators activate neutrophils, macrophages, and T lymphocytes, sustaining and amplifying inflammation (26).

Th17 cells are strongly linked to prognosis. IL-17 promotes anti-heart antibody production and progression to inflammatory DCM (iDCM) (6). Circulating Th17 levels are increased in myocarditis and DCM with left ventricle (LV) dysfunction and correlate with HF severity, being higher in NYHA III–IV than NYHA I–II patients (25). Accordingly, Th17 expansion is associated with failure of resolution and progression to chronic myocardial dysfunction and HF (25). Th17 expansion is accompanied by reduced Treg frequency compared with healthy controls, a process in which IL-6 is thought to play a central role (25). Tregs exert protective effects through several mechanisms, including promoting monocyte polarization toward M2 macrophages; which can enhance repair and improve LV function (6).

CD8+ T lymphocytes also contribute substantially to myocarditis through cytotoxic killing of infected cells via perforin and granzymes as well as by producing IFN-*γ* (6). While CD8 + responses support viral clearance, as suggested by more severe CVB3 myocarditis in murine models lacking CD8 cells or their receptors, progressing to chronic myocarditis (27), they can also contribute to myocardial injury and fibrotic remodeling (6). Finally, follicular helper T cells (Tfh) support B-cell activation and AHA production. Tfh cells produce IL-21, which drives B-cell expansion and differentiation (6).

## 4 Autoimmunity and molecular mimicry

### 4.1 Autoantibodies and pathogenic targets

Autoimmune myocarditis can evolve into chronic iDCM meeting Rose–Witebsky criteria, supporting the concept of organ-specific autoimmunity (10, 11, 27). AHAs are detected in up to ∼60% of patients with chronic cardiomyopathy and in some relatives (11). Several cardiac autoantigens have been identified, including *α*-myosin heavy chain (myosin 6), *β*-myosin heavy chain (myosin 7), the *β*1-adrenergic receptor (*β*1-AR), the M2 muscarinic receptor, and cardiac troponins, which are notable for potential pathogenic and prognostic roles (10, 27).

Immunization of experimental models with these autoantigens can induce phenotypes resembling human inflammatory cardiomyopathy (10). In addition, passive transfer of antibodies from mice immunized with cardiac myosin can lead to myocardial antibody deposition in recipient animals, promoting cardiomyocyte apoptosis and cardiomyopathy (10), consistent with organ-specific autoimmunity (10, 11). Importantly, not all AHAs are pathogenic; some may be epiphenomena or bystanders of immune-mediated injury (28). Anti-myosin (anti-CM) and anti–*β*1-AR antibodies may share a pathogenic mechanism via molecular mimicry between cardiac myosin and *β*1-AR. Both antibody specificities can target *β*1-AR and stabilize a constitutively active receptor conformation (13, 29), thereby promoting sustained adrenergic signaling. Once systolic dysfunction is established, chronic adrenergic overstimulation may be deleterious, inducing cardiomyocyte apoptosis (13). Apoptosis is mediated, at least in part, by increased cytosolic Ca2 + due to enhanced L-type Ca2 + channel conductance, driven by Gs signaling and PKA-dependent phosphorylation (30).

Autoantibodies against cardiac troponin I may also induce apoptosis. These antibodies have been reported to target enolase 1 (ENO1), a glycolytic enzyme that can also be expressed on the cell membrane as a dimer. Binding of anti–troponin I antibodies to ENO1 induced apoptosis through increased PTEN phosphorylation (a negative regulator of Akt) and reduced Akt phosphorylation (31). Autoantibodies against the M2 muscarinic receptor have been associated with risk of disease progression in myocarditis and early DCM. In murine ventricular cardiomyocytes, anti-M2R IgG reduced Ca2 + current amplitude. Although this would be expected to shorten action potential duration, action potentials were prolonged, resembling the effects reported for anti–*β*1-AR antibodies (32). Finally, in EMB-confirmed myocarditis, non–organ-specific antibodies such as antinuclear antibodies (ANA) have also been reported to have prognostic relevance (11).

### 4.2 Genetic predisposition

Genetic predisposition contributes to autoimmune myocarditis and inflammatory cardiomyopathy and helps explain detection of AHAs in asymptomatic relatives of affected patients (28). AHAs may have predictive value in relatives, in whom early echocardiographic abnormalities, such as mild ventricular dilation with preserved systolic function or early strain abnormalities, can be detected in ∼9%–21% of asymptomatic individuals. These findings support an association between positive serology and early disease stages. Baseline AHA positivity is more common among relatives who later show disease progression compared with those who do not (28). Autoimmune disease reflects a complex interaction between genetic susceptibility and environmental triggers. Genetic susceptibility may underlie positive serology in asymptomatic relatives (28). Associations have been reported with HLA-DR4 (13, 28, 33), which may influence both myocarditis development and risk of progression to DCM (33). Other positively associated alleles include HLA-DR12 and HLA-DR15. HLA-DQ1 has been implicated in predisposition to autoimmunity and myocarditis and has been reported to be more prevalent among patients with ICI-related myocarditis (33). Conversely, HLA-DR11 and DQB1*0301 have been reported to be as negatively associated (13). Non-HLA polymorphisms linked to autoimmune diathesis include CTLA-4, PD-1, and ICOS, which encode regulators of T-cell activity, including anergy and apoptosis (13, 33). Additional loci (e.g., Eam1 and Eam2) have been associated with myocarditis in the context of systemic autoimmune disease such as SLE or diabetes (33).

### 4.3 Transition to chronic inflammation and fibrosis

Genetic predisposition, impaired viral clearance, and persistence of autoimmune responses can prevent resolution of viral myocarditis, promoting chronic inflammation and fibrotic remodeling that may progress to iDCM. These processes are associated with persistently elevated circulating cytokine levels and continued immune cell accumulation within the myocardium (6). The Th17 axis is central to the sustained damage to the myocardium (6, 25). In murine models lacking IL-17, reduced MMP2 and MMP9 expression, decreased gelatinase activity, and reduced interstitial fibrosis have been reported (6). The CXCL4–CXCR3 axis also promotes fibrosis and is closely linked to TGF-*β* signaling. CXCL4 (also termed platelet factor 4, PF4) has relatively weak chemotactic activity but exerts strong pro-inflammatory and pro-fibrotic effects (34). In the myocardium, CXCL4 promotes fibrogenesis by activating fibroblasts through the TGF-*β*/Smad2/3 pathway, inducing expression of type I and III collagen and *α*-smooth muscle actin (*α*-SMA) (34).

## 5 Inflammation-Related biomarkers

### 5.1 Established markers

**Figure 3:**
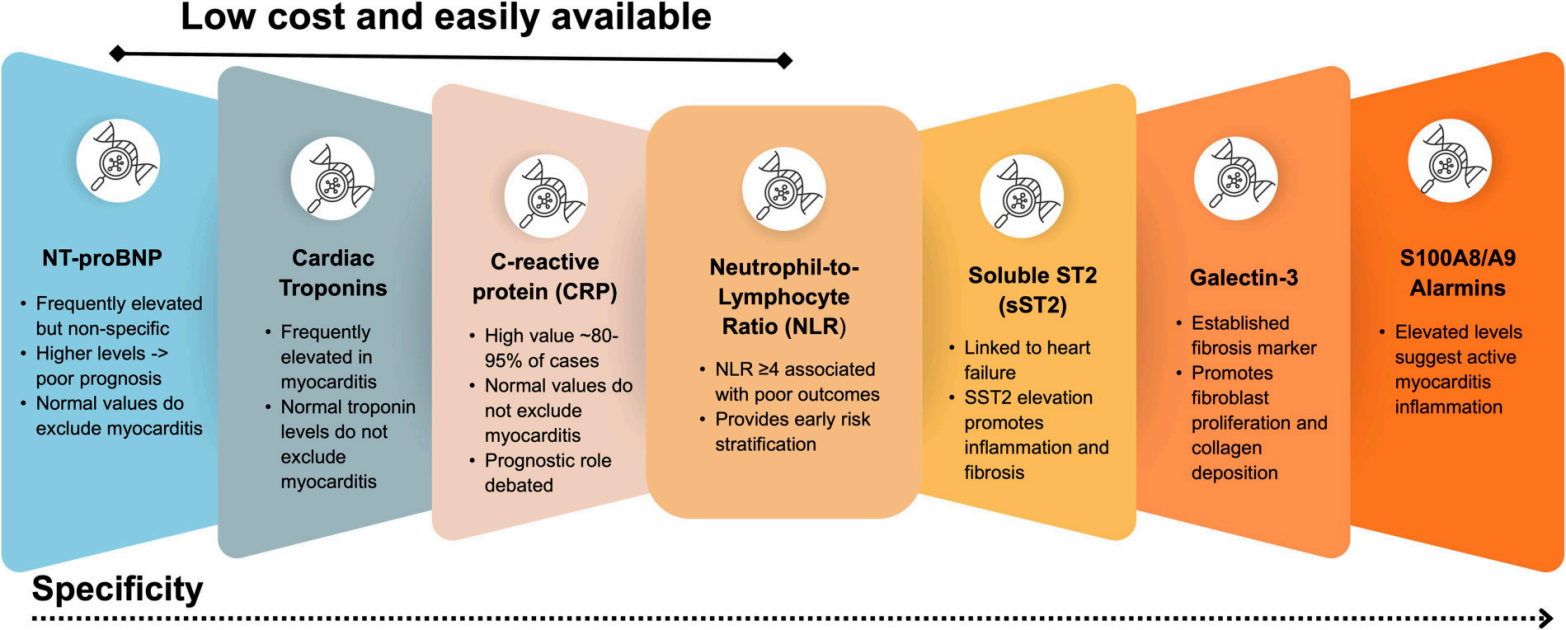
Conventional and emerging circulating biomarkers in myocarditis. The figure summarizes circulating biomarkers associated with myocardial injury, inflammation, immune activation, and fibrotic remodeling in myocarditis. Conventional biomarkers include troponins, C-reactive protein (CRP), and natriuretic peptides, while emerging inflammatory biomarkers include neutrophil-to-lymphocyte ratio (NLR), soluble ST2 (sST2), galectin-3, S100A8/S100A9. These biomarkers may contribute to diagnosis, inflammation-guided risk stratification, disease monitoring, and identification of patients at risk of adverse remodeling and heart failure progression.

Several circulating biomarkers have been investigated to support the diagnosis and risk stratification of myocarditis, reflecting myocardial injury, inflammatory activity, and hemodynamic stress (Figure 3). Cardiac troponins (cTnT and cTnI) are more sensitive than CK and CK-MB for detecting myocardial injury in infectious myocarditis (35). Troponins are frequently elevated and, together with clinical evaluation and imaging, can support the diagnosis (36). However, normal troponin values do not exclude myocarditis (12, 37). Troponin levels may normalize over time in patients with persistent disease (36). Admission peak troponin levels > 50 ng/L have been associated with adverse prognosis (37), although troponin levels correlate only modestly with left ventricular ejection fraction (LVEF) (10, 37).

C-reactive protein (CRP) is often elevated in AM (reported in ∼80%–95% of patients), but normal CRP does not exclude the diagnosis (37). Its prognostic role remains debated. In a retrospective study by Baritussio et al., CRP did not reliably identify patients at higher clinical risk (38, 39).

NT-proBNP is frequently elevated at presentation but may also be normal; therefore, it cannot exclude myocarditis and is not specific (37). Its diagnostic utility is limited, although higher levels have been associated with worse prognosis (35, 37).

### 5.2 Novel inflammatory markers

Beyond conventional inflammatory markers, increasing evidence supports the prognostic relevance of composite leukocyte-derived indices. In a large multicentre cohort study (observational clinical evidence) of patients with biopsy- or CMR-proven AM, the neutrophil-to-lymphocyte ratio (NLR) emerged as a robust predictor of adverse outcomes, including death and heart transplantation. Notably, an NLR ≥4 showed prognostic performance comparable to established high-risk clinical classifications and demonstrated superior discriminative ability in patients presenting with preserved LVEF, a subgroup traditionally considered at low risk (39, 40).

Soluble ST2 (sST2) has emerging evidence as a potential predictor of progression and prognostic marker in HF and in myocarditis (41). ST2 belongs to the IL-1 receptor family and functions as the receptor for IL-33. It exists in two major isoforms generated by alternative splicing: a transmembrane form (ST2L) and a soluble form (sST2) (42). IL-33 is expressed in stromal cells (including fibroblasts and myofibroblasts) and in endothelial cells and is released upon injury, functioning as an alarmin (43). IL-33 binding to ST2L mediates cardioprotective effects that are anti-inflammatory, anti-apoptotic, anti-hypertrophic, and anti-fibrotic (42). In contrast, sST2, released by cardiac fibroblasts and cardiomyocytes under myocardial stress, binds IL-33 with high affinity and acts as a decoy receptor, thereby neutralizing IL-33 signaling (41, 42). Consequently, elevated sST2 may favour inflammation and fibrotic remodeling and contribute to diastolic dysfunction (42).

Galectin-3 is another marker linked to fibrosis. Initially studied in oncology, galectin-3 participates in cellular adhesion, activation, chemotaxis, growth, differentiation, and apoptosis. Subsequently, it was implicated in HF, due to strong associations with chronic inflammation and interstitial fibrosis (42). Galectin-3 is expressed in various cell types and is particularly abundant in macrophages. Increased galectin-3 promotes fibroblast proliferation and collagen deposition, contributing to fibrotic remodeling (42).

Alarmins S100A8 and S100A9 have also been proposed as candidate biomarkers. Their serum levels may support diagnosis and monitoring in suspected myocarditis. They are released by activated neutrophils and monocytes/macrophages, and reported serum levels correlate with disease activity assessed by EMB (e.g., inflammatory cell counts using CD3, LFA-1, and MAC-1 markers) and with LVEF measured by echocardiography (44). From a clinical perspective, these biomarkers may contribute to early risk stratification, identify high-risk inflammatory phenotypes, and potentially guide therapeutic decision-making in selected patients.

### 5.3 Role of microRNAs

A large number of microRNAs (miRNAs) have been linked to immunopathological processes in viral myocarditis, mainly based on preclinical and translational evidence (33, 45). These miRNAs regulate transcription of proteins involved in inflammation and fibrosis (45). miR-21, miR-155, and miR-590 are increased in both human myocarditis and CVB3-induced murine models and promote inflammation and myocardial injury in viral and autoimmune myocarditis. In contrast, miR-221 and miR-222 appear protective, limiting viral replication and inflammation; their inhibition in experimental models increases viral load and inflammatory injury (45). The miRNAs also regulate NLRP3 inflammasome activation in viral myocarditis. miR-15 is markedly upregulated after CVB3 infection and represses NLRX1, thereby increasing IL-1β and IL-18 via enhanced NLRP3 activity. Conversely, miR-223 targets TRAF6 mRNA (encoding an E3 ubiquitin ligase required for IKK activation); inhibition of miR-223 enhances NF-*κ*B signaling and increases NLRP3 expression (17). Despite these mechanistic insights, clinical evidence supporting the diagnostic or therapeutic application of miRNAs in myocarditis remains limited.

## 6 Selected clinical scenarios

### 6.1 Fulminant myocarditis and unbalanced immune activation

Fulminant myocarditis is characterized by the abrupt onset of severe myocardial inflammation and represents the most dramatic and life-threatening presentation of AM. Clinically, it is defined by rapid development of profound left ventricular systolic dysfunction, frequently accompanied by cardiogenic shock, malignant ventricular arrhythmias, and advanced atrioventricular conduction disturbances, with historically high mortality rates despite aggressive supportive care (46, 47).

From an immunopathological perspective, fulminant myocarditis reflects a profound dysregulation of the immune response, in which excessive innate immune activation predominates over adaptive immune control. Early and massive activation of innate immune cells, particularly neutrophils, monocytes/macrophages, and NK cells, leads to an uncontrolled release of pro-inflammatory cytokines and chemokines, resulting in a cytokine storm–like syndrome. This exaggerated inflammatory response is often disproportionate to the degree of direct cardiomyocyte infection or injury and represents the primary driver of myocardial dysfunction. Mechanistically, excessive cytokine signaling (IL-1β, IL-6, TNF-α, interferons, and downstream NF-*κ*B activation) induces diffuse myocardial oedema, cardiomyocyte necrosis, and microvascular dysfunction. These processes are further amplified by endothelial activation and increased vascular permeability, which facilitate further immune cell infiltration and sustain a self-perpetuating inflammatory loop. Non-immune resident cardiac cells, including fibroblasts and endothelial cells, actively participate in this process by producing cytokines, chemokines, and adhesion molecules, thereby reinforcing immune-mediated injury.

Importantly, myocardial contractile failure in fulminant myocarditis is not solely attributable to structural damage (48). Pro-inflammatory cytokines exert direct negative inotropic effects, alter calcium handling, and disrupt electrical conduction, contributing to severe systolic dysfunction, arrhythmogenesis, and rapid hemodynamic collapse. This dissociation between inflammatory burden and irreversible myocardial damage may explain the potential for dramatic recovery observed in survivors once immune activation is promptly controlled (6).

Collectively, fulminant myocarditis exemplifies an inflammation-driven cardiac syndrome in which early recognition of immune dysregulation, timely immunomodulatory intervention and mechanical circulatory support, are critical determinants of survival (6, 48). Most of these mechanistic insights derive from preclinical and translational studies, with limited direct randomized clinical evidence.

### 6.2 ICI-related myocarditis

ICI–associated myocarditis represents an emerging and potentially life-threatening complication in the era of immune-based cancer therapy (5, 49, 50). By inhibiting key immune regulatory pathways such as cytotoxic T-lymphocyte–associated protein 4 (CTLA-4), programmed cell death receptor 1 (PD-1), and its ligand PD-L1, ICIs enhance T-cell–mediated antitumor immunity. However, they may also trigger uncontrolled immune activation against cardiac tissue (9, 51). Although the reported incidence of ICI-related myocarditis is relatively low (approximately 0.04%–1%), the condition is characterized by a disproportionately high mortality rate, ranging from 25% to 50% in early series, particularly when diagnosis and immunosuppressive therapy are delayed. Most cases occur early after treatment initiation, typically within the first 4–8 weeks, often after only one or two doses of therapy. Combination ICI regimens further increase both incidence and severity (51). The clinical presentation is heterogeneous and frequently nonspecific, encompassing dyspnoea, chest pain, palpitations, and fatigue. Importantly, severe manifestations, including cardiogenic shock, malignant ventricular arrhythmias, and high-grade atrioventricular block, may occur abruptly and are not reliably predicted by LVEF. Indeed, a substantial proportion of patients present with preserved or only mildly reduced LVEF, underscoring the limitations of conventional cardiac risk stratification in this setting (52). From a pathophysiological perspective, ICI-related myocarditis shares striking similarities with autoimmune and virus-negative inflammatory myocarditis. Histopathological studies consistently demonstrate dense myocardial infiltration by activated CD4⁺ and CD8⁺ T lymphocytes, frequently accompanied by macrophages, with evidence of T-cell clonal expansion shared between tumour and cardiac tissue. Experimental models further support a critical cardioprotective role of the PD-1/PD-L1 axis, as genetic disruption of this pathway leads to autoimmune DCM and myocardial inflammation (9, 51). These mechanisms translate into a clinical phenotype in which immune dysregulation, rather than direct cardiomyocyte injury alone, appears to drive disease severity and prognosis. Therefore, biomarkers reflecting systemic inflammatory activation may provide incremental prognostic information beyond traditional markers of myocardial damage or dysfunction. The frequent dissociation between inflammatory burden, troponin elevation, and ventricular function mirrors observations in other forms of AM. It reinforces the concept that immune-mediated injury can precede overt structural or functional deterioration (9, 51). Collectively, ICI-associated myocarditis exemplifies an inflammation-driven cardiac syndrome in which early recognition of immune activation and prompt immunomodulatory intervention are crucial. This paradigm supports integrating inflammatory biomarkers and immune-based risk stratification tools into clinical decision-making and provides a strong rationale for exploring targeted anti-inflammatory therapies in selected high-risk patients.

### 6.3 COVID-19 and vaccine-associated myocarditis

SARS-CoV-2 is an enveloped, single-stranded RNA virus belonging to the Coronaviridae family. The spike protein is central to viral entry and consists of S1 and S2 subunits; S1 binds ACE2 receptors on host cells (53). Although infection is primarily respiratory, systemic manifestations occur because ACE2 is expressed in multiple organs (53, 54). The heart can be affected, as ACE2 is expressed in cardiomyocytes and pericytes and is also present on macrophages, fibroblasts, and endothelial cells (54).

Detection of SARS-CoV-2 RNA by PCR in myocardial tissue from autopsies supports the possibility of direct cardiac infection (52). One proposed mechanism is direct viral entry and replication in cardiomyocytes (53). Another hypothesis emphasizes immune-mediated injury driven by excessive inflammation (“cytokine storm”) (53, 54). Several mechanisms may contribute, including molecular mimicry between spike protein and cardiac autoantigens (6). Downregulation of ACE2 may also contribute by suppressing the protective Ang- (1–7)/Mas receptor axis (6, 55). In addition, complement activation, TLR4 signaling, and inflammasome activation may contribute to dysregulated cytokine release (55). Endothelial dysfunction represents another important mechanism. It may be driven by direct pericytes infection and can promote the release of inflammatory cytokines and microvascular thrombosis in the coronary circulation (6, 53).

Although uncommon, myocarditis has been described after mRNA vaccination against SARS-CoV-2 (27). Proposed mechanisms include molecular mimicry between the spike protein and cardiac myosin, as well as cytokine-mediated inflammatory pathways similar to those described above (54). Clinically, SARS-CoV-2–associated myocarditis ranges from mild disease to cardiogenic shock, whereas vaccine-associated myocarditis is typically milder and has a favourable prognosis (53).

## 7 Therapeutic implications

### 7.1 Anti-inflammatory and immunosuppressive strategies

The inflammatory pathways described above provide the biological rationale for both current and emerging therapeutic strategies in myocarditis. Because a substantial proportion of AM cases recover spontaneously, management should be tailored to clinical severity, disease trajectory, and etiologic definition based on endomyocardial biopsy with viral PCR (2). An overview of the principal immune mechanisms, their associated biomarkers, and potential therapeutic implications is provided in Table 1. In hemodynamically stable patients with HF, guideline-directed medical therapy (GDMT) remains the cornerstone of management (12, 56). Current guideline recommendations emphasize supportive therapy and the selective use of immunosuppression based on etiologic and histological characterization, highlighting the central role of endomyocardial biopsy in guiding treatment decisions. Beyond their established hemodynamic effects, several HF agents exert pleiotropic actions that may indirectly modulate inflammatory and remodeling pathways, including attenuation of oxidative stress, cytokine signaling, and extracellular matrix turnover, thereby providing a mechanistic rationale for their use in inflammatory cardiomyopathy (2, 11).

**Table 1:** Key inflammatory pathways in myocarditis: mechanistic insights and therapeutic implications.

Immune pathway/axis	Key cells involved	Principal mediators	Pathophysiological role	Associated biomarkers	Therapeutic implications	Level of evidence	References
Innate immune activation/PRRs	Macrophages, dendritic cells, neutrophils	TLRs, RIG-I, MDA5, NF-*κ*B	Early recognition of PAMPs/DAMPs; initiation of inflammatory cascade	CRP, NLR, S100A8/A9	Supportive care; early immunomodulation in selected cases	Preclinical + translational	(6, 14–17)
NLRP3 inflammasome/IL-1 axis	Macrophages, cardiomyocytes	NLRP3, caspase-1, IL-1β, IL-18	Amplification of myocardial inflammation; pyroptosis; adverse remodeling	CRP, IL-1–related signatures	IL-1 blockade (e.g., anakinra); ongoing clinical trials	Preclinical + early clinical	(17, 57, 59, 60)
Th1-mediated immunity	CD4⁺ Th1 cells, macrophages	IFN-*γ*, IL-12, TNF-α	Viral clearance; macrophage activation; balance between protection and injury	Troponins, cytokine profiles	Generally protective; excessive activation may require immunosuppression	Preclinical + observational	(6, 24)
Th17/Treg imbalance	Th17 cells, Tregs, monocytes	IL-17, IL-6, IL-23, GM-CSF, TGF-β	Sustained inflammation; promotion of fibrosis; progression to iDCM	NLR, IL-17–related profiles	Emerging targets (e.g., anti–IL-17 strategies); experimental	Preclinical + observational	(6, 23, 25, 26)
IL-33/ST2 axis	Fibroblasts, cardiomyocytes, endothelial cells	IL-33, ST2L, soluble ST2 (sST2)	Loss of cardioprotective IL-33 signaling; promotion of fibrosis and diastolic dysfunction	sST2	Risk stratification; potential future therapeutic modulation	Observational	(41–43)
Fibrotic remodeling pathways	Fibroblasts, macrophages	Galectin-3, TGF-β, MMPs	Interstitial fibrosis; adverse ventricular remodeling	Galectin-3	Identification of patients at risk of chronic dysfunction	Observational	(24, 34, 35, 42)
Adaptive cytotoxic immunity	CD8⁺ T cells, NK cells	Perforin, granzymes, IFN-γ	Clearance of infected cardiomyocytes; contribution to myocardial injury	Troponins	Immunosuppression in selected immune-mediated forms	Early clinical (non-RCT) + emerging RCT	(6, 10, 46)
Immune checkpoint dysregulation (ICI-related)	CD4⁺/CD8⁺ T cells, macrophages	PD-1/PD-L1, CTLA-4 pathways	Uncontrolled T-cell activation; fulminant immune-mediated injury	Troponins, NLR	High-dose corticosteroids; early aggressive immunosuppression	Observational + translational	(5, 49, 51, 52)

By contrast, anti-inflammatory and immunosuppressive therapies require a more selective and context-dependent approach. Non-steroidal anti-inflammatory drugs (NSAIDs) should not be considered routine therapy for myocarditis and are mainly justified in the presence of associated pericarditis, where anti-inflammatory dosing may alleviate chest pain and inflammatory symptoms, with appropriate gastroprotection (2). Broad empirical immunosuppression in unselected AM remains controversial, as suppression of host immune responses may be ineffective or potentially harmful when viral replication persists. Accordingly, current recommendations emphasize excluding active infection, ideally by endomyocardial biopsy with viral PCR, before initiating immunosuppressive treatment. Nevertheless, in severe or rapidly evolving presentations, therapeutic decisions may precede complete etiologic characterization, highlighting the tension between mechanistic precision and clinical urgency (57).

Within this framework, intravenous immunoglobulins (IVIG) occupy an uncertain position in the treatment of adult myocarditis. Although high-quality randomized trials are lacking, observational data have suggested potential benefit in selected cohorts, including improved transplant-free survival. Consequently, guidelines refrain from firm recommendations and underscore the need for adequately powered randomized studies. IVIG remains more commonly employed in paediatric myocarditis, reflecting differences in disease biology and clinical practice (2).

Similarly, the use of corticosteroids in AM requires careful clinical judgment. Routine empirical administration in unselected cases is not supported by current evidence, reflecting the dual role of inflammation, which may be either adaptive or maladaptive depending on timing, intensity, and etiology. However, corticosteroids represent a cornerstone of therapy in well-defined immune-mediated contexts, including immune checkpoint inhibitor–associated myocarditis, eosinophilic myocarditis, and selected biopsy-proven autoimmune forms such as giant cell myocarditis. Beyond these established indications, the ongoing MYocarditis THerapy with Steroids (MYTHS) trial is designed to clarify whether early high-dose corticosteroid therapy can improve outcomes in patients with fulminant myocarditis or AM complicated by severe left ventricular dysfunction, targeting those at highest inflammatory and clinical risk (58).

In patients presenting with fulminant myocarditis or AM complicated by hemodynamic compromise, therapeutic priorities extend beyond pharmacological immunomodulation. Early escalation of care, including prompt multidisciplinary Shock Team evaluation and timely consideration of temporary mechanical circulatory support, is essential to stabilize patients, preserve end-organ perfusion, and create a therapeutic window for diagnostic clarification and targeted intervention (2, 10).

### 7.2 Emerging targeted therapies

Growing mechanistic and clinical evidence supports inflammation as a key driver of myocardial injury and adverse outcomes in myocarditis, providing a strong rationale for targeted immunomodulatory strategies. Beyond pathogen-directed approaches, several therapies aimed at specific inflammatory pathways are currently under investigation.

Preclinical studies have shown that anti–coxsackievirus and adenovirus receptor (CAR) antibodies can attenuate both acute myocardial injury and chronic remodeling in murine models of CVB3-induced myocarditis, highlighting the potential of upstream antiviral–immunomodulatory interventions (10).

Among cytokine-targeted therapies, inhibition of the IL-1 axis has emerged as a particularly promising strategy. IL-1 plays a central role in inflammasome-mediated myocardial inflammation, linking innate immune activation to cardiomyocyte dysfunction and adverse remodeling. Clinical studies (mainly small-scale and non-randomized) have demonstrated that anakinra, a recombinant IL-1 receptor antagonist (IL-1Ra), can reduce myocardial inflammation and improve systolic function in patients with AM and fulminant myocarditis (11). Consistently, experimental models have shown that IL-1β neutralization prevents progression toward chronic viral myocarditis by limiting sustained inflammation and interstitial fibrosis (10).

On this basis, anti–IL-1 strategies (anakinra, rilonacept, canakinumab) and colchicine are biologically plausible, although robust human myocarditis data remain limited (2).

In this context, the ARAMIS trial, which provides randomized clinical trial evidence, constitutes a pivotal step, not for demonstrating definitive efficacy, but for highlighting the limitations of non-enriched trial designs in myocarditis. The neutral results observed in a largely low-risk population emphasize that therapeutic failure may reflect inappropriate patient selection rather than a lack of biological relevance of the target. These findings strongly support a shift toward inflammation-guided therapeutic approaches (59, 60). Parallel efforts have focused on adaptive immune pathways, particularly the imbalance between pro-inflammatory Th17 responses and regulatory T-cell–mediated immune tolerance. A clinical trial of secukinumab (anti–IL-17A) has been proposed to blunt Th17-driven immune activation. In parallel, cellular therapies based on adoptive transfer or expansion of regulatory T cells (Tregs) represent an experimental but conceptually attractive strategy to restore immune tolerance and counteract chronic inflammation (10).

## 8 Conclusion

Myocarditis is an inflammation-driven disease in which dysregulated immune responses critically determine clinical outcomes. A deeper mechanistic understanding of immunopathogenesis is essential to enable the development of targeted therapies, refine risk stratification, and move beyond conventional diagnostic paradigms toward earlier and more specific tools. In this context, emerging biomarkers, including miRNAs and alarmins such as S100A8/S100A9, as well as NLR, sST2 and galectin-3, may support disease monitoring and identify patients at risk of adverse remodeling. However, the level of evidence supporting these approaches remains heterogeneous, underscoring the need for dedicated, mechanism-driven clinical trials and the development of inflammation-guided precision strategies in myocarditis.
